# Three-dimensional object geometry of mitochondria-associated signal: 3-D analysis pipeline for two-photon image stacks of cerebrovascular endothelial mitochondria

**DOI:** 10.1152/ajpheart.00101.2024

**Published:** 2024-03-22

**Authors:** Christopher R. Gonzales, Eric N. Moca, Partha K. Chandra, David W. Busija, Ibolya Rutkai

**Affiliations:** ^1^Department of Pharmacology, Tulane University School of Medicine, New Orleans, Louisiana, United States; ^2^Tulane Brain Institute, Tulane University, New Orleans, Louisiana, United States

**Keywords:** endothelial mitochondria, three-dimensional object geometry, two-photon images

## Abstract

Increasing evidence indicates the role of mitochondrial and vascular dysfunction in aging and aging-associated pathologies; however, the exact mechanisms and chronological processes remain enigmatic. High-energy demand organs, such as the brain, depend on the health of their mitochondria and vasculature for the maintenance of normal functions, therefore representing vulnerable targets for aging. This methodology article describes an analysis pipeline for three-dimensional (3-D) mitochondria-associated signal geometry of two-photon image stacks of brain vasculature. The analysis methods allow the quantification of mitochondria-associated signals obtained in real time in their physiological environment. In addition, signal geometry results will allow the extrapolation of fission and fusion events under normal conditions, during aging, or in the presence of different pathological conditions, therefore contributing to our understanding of the role mitochondria play in a variety of aging-associated diseases with vascular etiology.

**NEW & NOTEWORTHY** Analysis pipeline for 3-D mitochondria-associated signal geometry of two-photon image stacks of brain vasculature.

## INTRODUCTION

The role and therapeutic potential of physiological and pathological mitochondrial processes represent an emerging field. We and others have shown that mitochondria are essential in normal cell function (1–4) and respond to various stress/stimuli (1, 5–7). Despite diverse morphology and metabolic functions among discrete cell types, their dynamic nature is alike. Ongoing and balanced changes in mitochondrial shape, size, or location demonstrate their adaptability to meet the energetic needs of cells. These dynamic processes result in either mitochondrial fragmentation or elongation via fission or fusion, respectively (8, 9). Mitochondrial fission and fusion are initiated, coordinated, and executed by specialized and well-conserved proteins, including the dynamin-related protein 1 (DRP-1) and fission 1 (FIS1) as well as mitofusin 1/2 (Mfn1/2) and optic atrophy 1 (OPA-1), respectively (7, 10–12). The recruited cytosolic DRP-1 and accessory proteins facilitate the constriction of mitochondrial membranes (fission), whereas Mfn1/2 and OPA-1 mediate outer and inner membrane fusions, respectively (7, 13). Under healthy conditions, mitochondrial fission and fusion are in balance and play an important role in the distribution of mtDNA and organelles during cell division, two of the determinants of cellular function (14, 15). Posttranslational modifications or increased/decreased expression, and/or activity of these proteins can shift the balance in either direction (16–18). Although mitochondria are essentially involved in all aspects of cellular function, one of their fundamental roles is to serve as energy sources. Shifts in mitochondrial shape/morphology are often accompanied by changes in mitochondrial energetics and architecture (19, 20). Elongated organelles (e.g., decreased fission or increased fusion) are frequently linked to better energetics and vice versa. Therefore, it is important to note that mitochondrial dysfunction is often an early sign of disease pathology in type 2 diabetes, insulin resistance, or Alzheimer’s disease (AD) (21–24). In addition, we and others have detected aging-related profound changes in the brain vasculature. Specifically, we found significantly decreased protein abundance of mitochondrial proteins including mitochondrial respiratory complex subunits or glycolysis-related proteins in isolated brain microvessels from aged mice compared with young (25–27). Others have reported a significantly decreased mitochondrial respiration and reduced ATP production in microvessels from aged mice as well as altered fuel dependency in aged endothelial cells compared with young (28, 29). Although past observations regarding changes in structure and/or function were frequently restricted in space and time, the methods we present in this paper provide valuable information about mitochondrial dynamics. We provide a protocol for multidimensional analysis of mitochondria-associated signal in three-dimensional (3-D) image stacks that can reveal information about the complexity and dynamics of the in vivo endothelial mitochondrial network, imaged multiple times over the course of the experiment.

## METHODS

### Analysis of Mitochondria-Associated Signal

We developed a new protocol for the morphological analysis of the mitochondria-associated Dendra2 signal (mito-Dendra2) in 3-D two-photon image stacks, advancing our previously reported method using maximum intensity projected two-dimensional (2-D) images for area analysis. We reused 3-D image stacks of three mito-Dendra2 expressing female mice that were taken with a two-photon microscope at four different time points and reported in Rutkai et al. (30). Briefly, images were obtained in the first-generation offspring of female PhAM-floxed [Jackson Laboratory: B6;129S-Gt(ROSA)26Sortm1(CAG-COX8A/Dendra-2)Dcc/J, Stock No. 018385] and male Tie2-Cre mice [B6.Cg-Tg(Tek-cre)1Ywa/J; Stock No. 008863] (31, 32). Five randomly selected images were used per mice and analyzed across four consecutive imaging time points (T-0, T-1, T-2, T-3).

**Figure 1.**
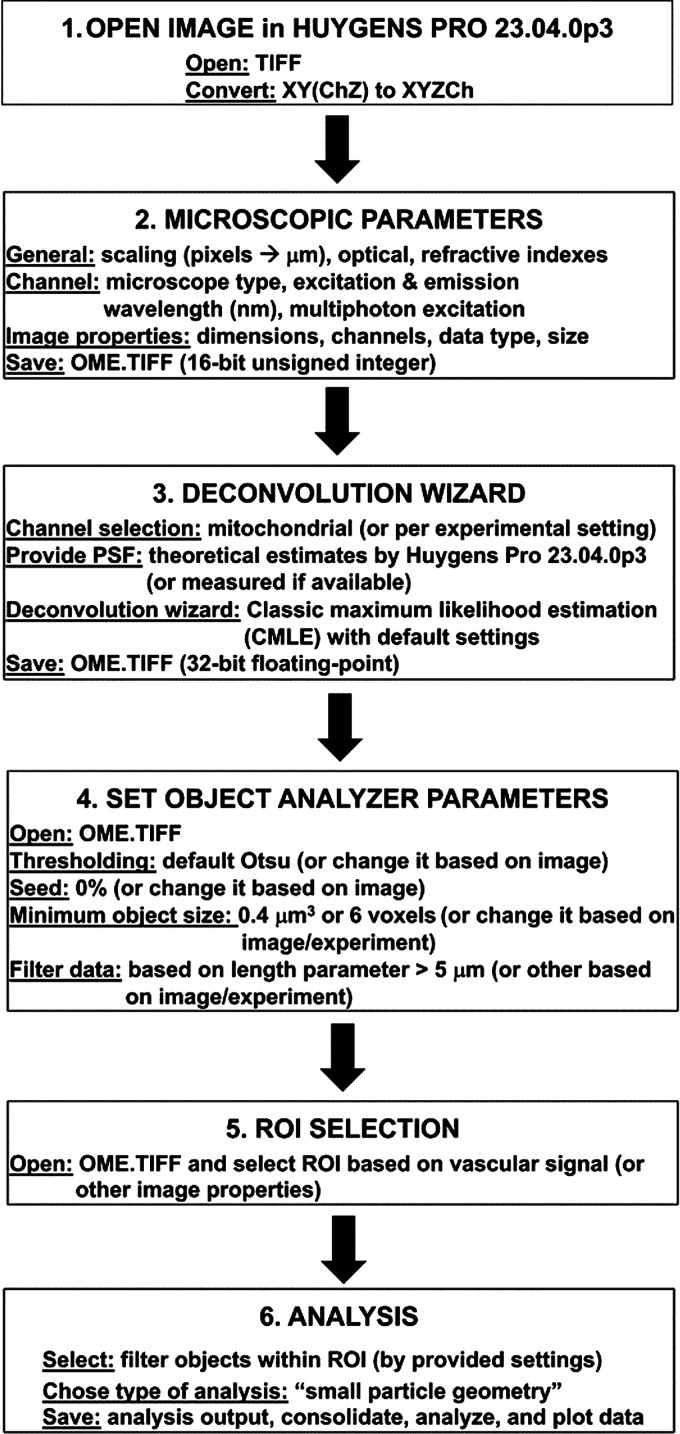
Schematic of three-dimensional (3-D) mitochondrial analysis workflow.

We used Huygens Pro 23.04.0p3 software for the morphological analysis of mito-Dendra2 signal in 3-D image stacks (Scientific Volume Imaging, The Netherlands; http://svi.nl). Original TIFF images were opened in Huygens Pro 23.04.0p3 and converted from XY(ChZ) to XYZCh to represent two channels, one for the mitochondrial signal and the other for the vascular signal. “Image statistics,” including the voxel size of *x/y/z*-dimensions, number of frames, channels, type of data, etc., were then checked and verified in Huygens Pro 23.04.0p3. Microscopic parameters were edited to reflect the sampling intervals (scaling), optical parameters (specific to the objective, used for imaging, e.g., 0.8 NA), refractive indexes for lens immersion (specific to the objective and imaging medium, used during imaging, e.g., water 1.338 and 1.398 for the imaging medium, considering the presence of water, glass coverslip, and brain tissue during imaging), or the channel parameters (e.g., excitation and emission wavelengths), and the type of excitation (e.g., 2 photon). Images were then saved as OME.TIFF 16-bit unsigned integer. After inputting and checking the image properties, images were deconvoluted on the mitochondrial channel only using Huygens Pro 23.04.0p3 Version deconvolution wizard and classic maximum likelihood estimate (CMLE) with default settings. We applied a theoretical PSF correction to the images, calculated by the software, and the images were then saved in OME.TIFF 32-bit floating-point format. These deconvoluted images were loaded in the Huygens Pro 23.04.0p3 Object Analyzer to determine mito-Dendra2 signal within the vasculature-associated regions of interest (ROIs), using the default Otsu thresholding, 0.4 μm^3^ as a minimum object size, and seed setting of 0%. Afterward, small particle geometry was selected for analysis type where objects were filtered based on their length, using the object length parameter (>5 μm) (Fig. 1). We measured 13 object geometry or mito-Dendra2 signal parameters using the Huygens Pro 23.04.0p3 Object Analyzer, including voxel volume, isosurface volume, object surface, length, smallest and largest width of the principal box, corrected lateral width, axial width, roughness sphericity, axial sphericity, axial sphericity of the principal box, axial and lateral aspect ratios. These parameter definitions and calculations can be accessed on the Huygens SVI website: https://svi.nl/ObjectAnalyzerExpertTutorial. Image processing and analysis were done by one laboratory personnel, and the compiled data and the results of the analysis were then checked by another investigator.

### Measurement of Vessel Diameter

We used the macro VasoMetrics (33) and ImageJ Fiji (34) to analyze vessel diameter in the original TIFF image stacks. The https://github.com/mcdowellkonnor/ResearchMacros website was used to download the code, and “VasoMetrics.ijm” was then saved to the Fiji macros folder. Scaled and deinterleaved images, containing the vascular signal, were opened with VasoMetrics and used to generate the maximum intensity projected images. We followed instructions for diameter measurements, provided by McDowell et al. (33). Briefly, after drawing the center lines, we used five cross lines/vessel segments or ROIs. Diameter measurements were done by two laboratory members.

### Statistical Analysis

#### Mito-Dendra2 signal geometry.

**Figure A1.**
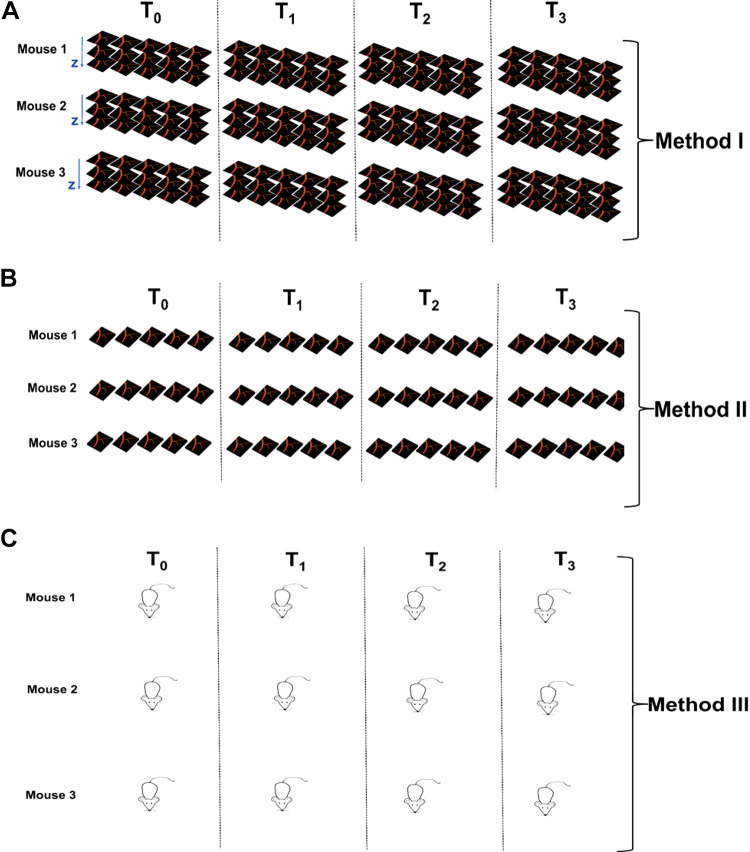
Schematics of data organization methods. *A*: for *Method_I*, data were used for statistical analysis, representing the individual mito-Dendra2 signal within each frame of three-dimensional image stacks/time point with object numbers: *N*_T-0_ = 3,123; *N*_T-1_ = 2,586; *N*_T-2_ = 2,111; and *N*_T-3_ = 3,363, 5 image stacks/mouse, 15 images stacks/time point, a total of 60 images originated from three mice. T_0_–T_3_ includes four consecutive imaging time points; “z” represents the “z steps” within image stacks. *B*: for *Method_II*, data were organized by image medians, resulting in 15 medians/time point. *C*: for *Method_III*, medians by mouse were used, resulting in 3 medians/time point.

Randomly selected five images/mouse/time point originated from three mice with the total number of 60 image stacks used for object (mito-Dendra2 signal) geometry analysis (3 mice × 5 image stacks per mouse × 4 consecutive imaging time points = 60). Data were organized in three ways: *Method I* (M_I) to show the individual objects (mito-Dendra2 signal) within each *Z*-stacks/time point (object numbers: *N*_T-0_ = 3,123; *N*_T-1_ = 2,586; *N*_T-2_ = 2,111; *N*_T-3_ = 3,363) (see appendix, Fig. A1*A*). *Method_II* (M_II) shows the median of each image/time point (15/time point) (see appendix, Fig. A1*B*). *Method_III* (M_III) uses the medians by mouse/time point (3/time point) (see appendix, Fig. A1*C*).

**Figure 2.**
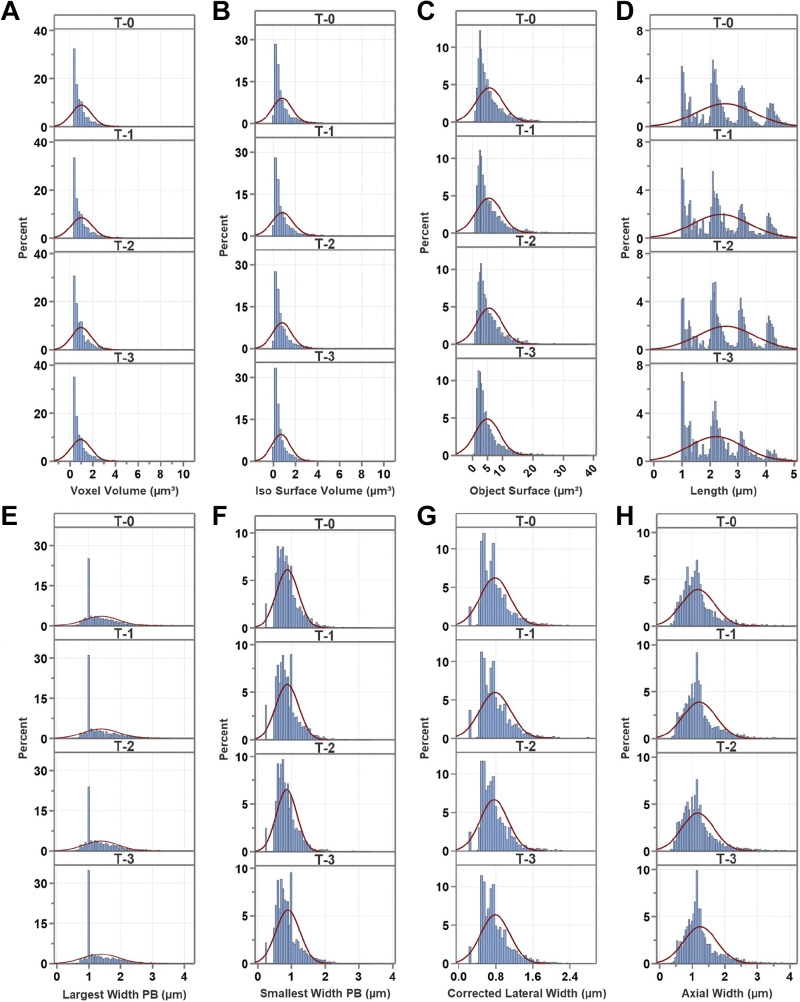
Data histograms for individual parameters. Histograms show data distribution for voxel volume (μm^3^; *A*), isosurface volume (μm^3^; *B*), object surface (μm^2^; *C*), length (μm; *D*), largest width of the principal box (PB; μm; *E*), smallest width of the PB (μm; *F*), corrected lateral width (μm; *G*), and axial width (μm; *H*). Normal distribution is projected in red over the histogram bins to show departure from normality. Consecutive imaging time points are denoted by T-0 (*N* = 3,123), T-1 (*N* = 2,586), T-2 (*N* = 2,111), and T-3 (*N* = 3,363). *N*_images_ = 60, originated from three mice.

**Figure 3.**
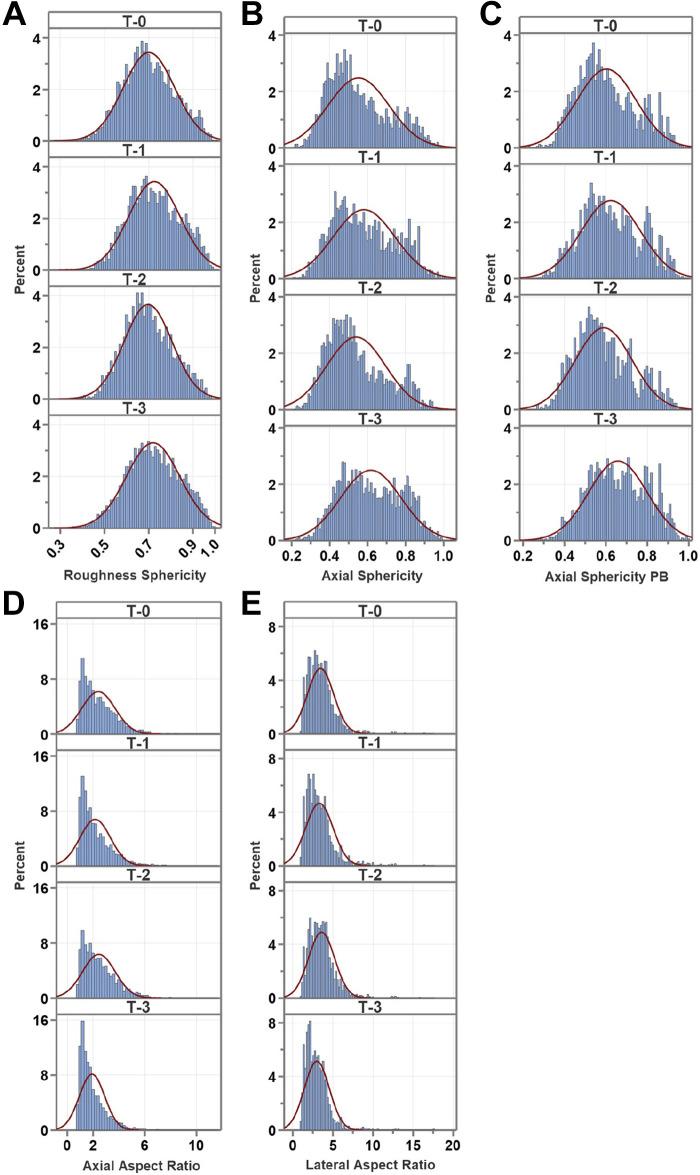
Data histograms for derived parameters. Histograms show data distribution for three different object sphericity parameters: roughness sphericity (*A*), axial sphericity (*B*), and axial sphericity of the principal box (PB; *C*), as well as the axial (*D*) and lateral (*E*) aspect ratios. Red curve over histogram bins represents theoretical Gaussian distribution indicating data departure from normality. Consecutive imaging time points are denoted by T-0, T-1, T-2, and T-3. *N*_images_ = 60, originated from three mice.

**Figure 4.**
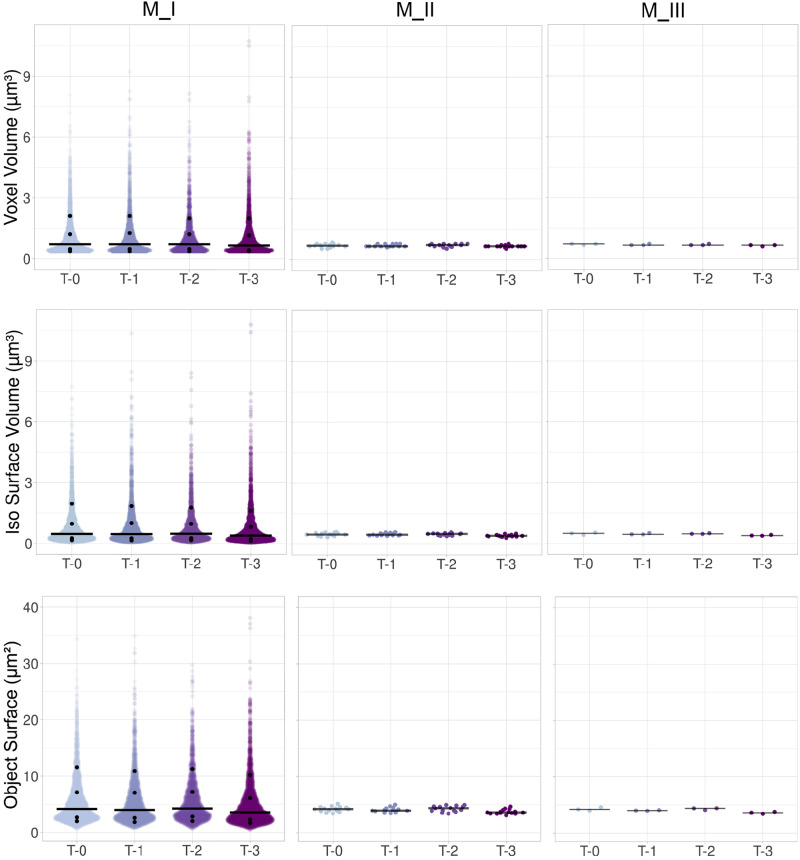
Scatter plots for voxel volume, isosurface volume, and surface. Data are shown as jittered dots for *Methods_I, _II*, and *_III* (M_I, M_II, and M_III, respectively), indicated by columns. Rows denote the voxel volume (μm^3^), isosurface volume (μm^3^), and object surface (μm^2^). Black horizontal bars represent the median. Medians for the different variables are listed in appendix in Table A1. Jittered plots for M_I also show the 10th, 25th, 75th, and 90th percentiles marked by black dots and listed in appendix in Table A3. Observation numbers are *N*_T-0_ = 3,123; *N*_T-1_ = 2,586; *N*_T-2_ = 2,111; and *N*_T-3_ = 3,363; for M_I from 60 images; and *N* = 15 per time points for M_II; and *N* = 3/time points for M_III.

**Figure 5.**
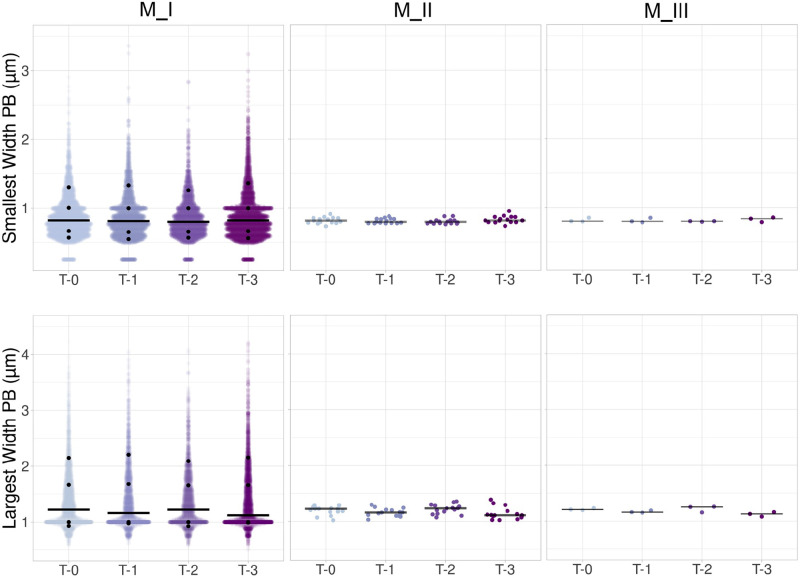
Scatter plots for the smallest and the largest width of the principal box (PB). Data are presented as jittered dots with the median (solid black bar) for the smallest width of the PB (in μm) and for the largest width of the PB (in μm) for three data analysis approaches. Tables A1 and A3 contain the medians for all the methods and the 10th, 25th, 75th, and 90th percentiles for M_I plots. Observation numbers for M_I were *N*_T-0_ = 3,123; *N*_T-1_ = 2,586; *N*_T-2_ = 2,111; and *N*_T-3_ = 3,363 originated from 60 images; *N* = 15 per time points for M_II and *N* = 3/time points for M_III.

**Figure 6.**
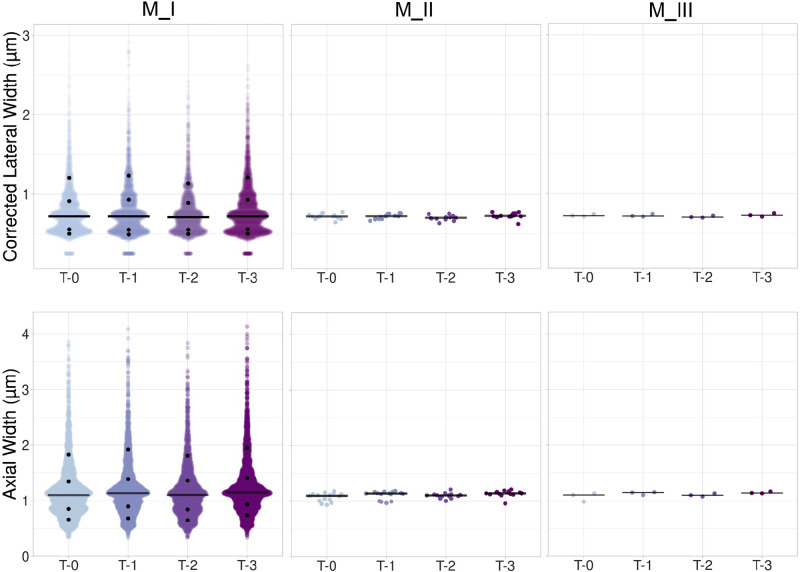
Scatter plots for corrected lateral width and axial width. Jittered dot plots with median (black bar) show data for the corrected lateral width (in μm) and for the axial width (in μm) for M_I through M_III analysis approaches. Black dots on M_I plots indicate the 10th, 25th, 75th, and 90th percentiles. Medians and percentiles are listed in Tables A1 and A3, respectively. Observations for M_I were *N*_T-0 _= 3,123; *N*_T-1_ = 2,586; *N*_T-2_ = 2,111; and *N*_T-3_ = 3,363 from 60 images; *N* = 15 per time points for M_II and *N* = 3/time points for M_III.

**Figure 7.**
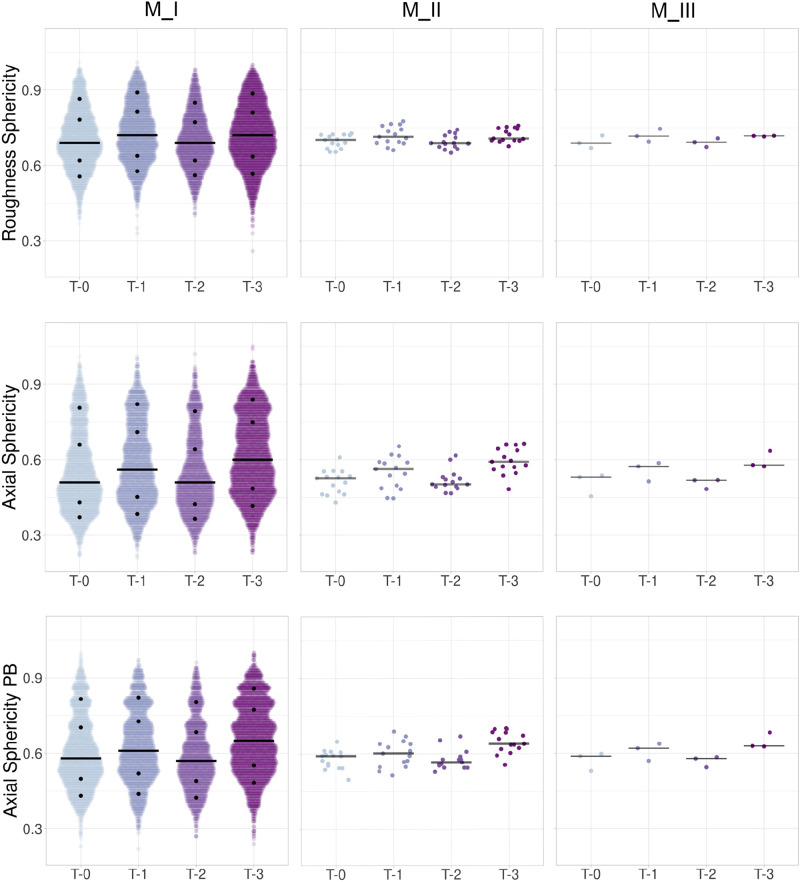
Scatter plots for sphericity indexes. Data are shown as jittered dots with the median represented by a black bar. Plots are shown for roughness sphericity, axial sphericity, and axial sphericity of the principal box (PB) for the three data analysis approaches. The median values are listed in Table A1. M_I data plots are presented with the 10th, 25th, 75th, and 90th percentiles as black dots that are listed in Table A3. Observation numbers for M_I include 3,123; 2,586; 2,111; and 3,363 for T-0 through T-3, respectively, from 60 images. Observations for M_II include *N* = 15 per time point, and for M_III, *N* = 3 per time point.

**Figure 8.**
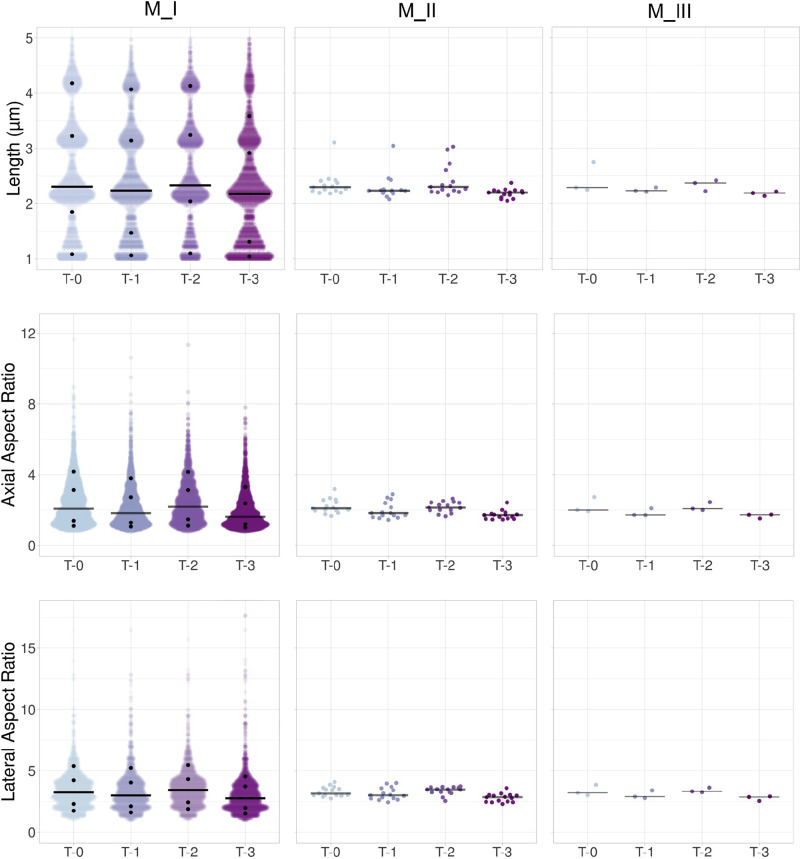
Scatter plots of morphological parameters for length and axial and lateral aspect ratios. Jittered plots show the data for length (in µm) and axial and lateral aspect ratios for M_I–M_III approaches. Black bars represent the medians listed in Table A1. Black dots on M_I plots denote the 10th, 25th, 75th, and 90th percentiles. Observations for the consecutive imaging time points for M_I were *N*_T-0_ = 3,123; *N*_T-1 _= 2,586; *N*_T-2_ = 2,111; and *N*_T-3_ = 3,363 from 60 images, originated from three mice; M_II, *N* = 15 per time point, and for M_III, *N* = 3 per time point.

Statistical analysis was conducted on M_I: data distribution was compared with a theoretical normal distribution using histograms, generated in Minitab Version 21.4.1 (Minitab, LLC, 2023; https://www.minitab.com) (Figs. 2 and 3). The one-sample Kolmogorov–Smirnov test with Lilliefors Significance Correction and the Shapiro–Wilk test revealed that all variables significantly deviated from normal distribution (*P* < 0.001; IBM SPSS Statistics for Windows, Version 27.0; IBM, Armonk, NY). Homogeneity of variance was assessed for all variables using the Brown–Forsythe Test (Levene’s test based on the median in IBM SPSS Version 27). We found that all variables showed non-unequal variances (*P* < 0.03) except for the largest width of the principal box and the axial width that had homogeneous variances (*P* = 0.659 and *P* = 0.807, respectively). The Kruskal–Wallis test (35) followed by the Dwass–Steel–Critchlow–Fligner post hoc pairwise comparisons (jamovi Version 2.4.11) were used to test for differences among mito-Dendra2 geometry variables (The jamovi project 2023; www.jamovi.org). Data analysis was also done with the multiresponse permutation procedure (MRPP) (36) using the Blossom Statistical Package Version W2008.04.02 (https://www.sciencebase.gov) with the Holm–Bonferroni *P* value correction (37). We used the open-source “Plots of Data” web tool to prepare jittered data plots with the median and selected percentiles (https://huygens.science.uva.nl/PlotsOfData/) (Figs. 4, 5, 6, 7, and 8) (38).

### Vascular Parameter

**Figure 9.**
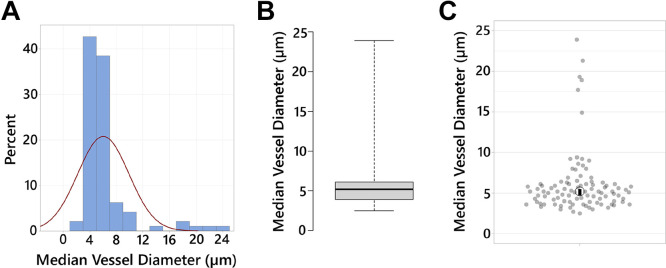
Vascular parameters. *A*: distribution of vessel diameter measurements (in μm) is shown where the red curve denotes normal Gaussian distribution. *B*: box plot shows median vessel diameter (96 data points) where whiskers indicate the minimum and maximum values. *C*: scatter plot shows same data as median with a 95th-percentile confidence interval. Diameters were measured in T-0 images only, *N*_images_ = 15, originated from three mice.

Diameter measurements were done on T-0 images only, for a total of 15 images (5 images/mouse). The reported diameter measurements represent the median of five diameter measurements completed independently by two laboratory members. For larger and/or penetrating vessels, the diameter was measured once by both laboratory members. These images were obtained using a 40× objective with a 3× digital zoom; thus, images contained short vessel segments. Therefore, we report diameter measurements for descriptive purposes without further analysis. Diameter measurements are presented via histogram (Fig. 9*A*), box plot (Fig. 9*B*), and scatter plot (Fig. 9*C*) generated using Minitab 21.4.1, “BoxPlotR” (39), and “PlotsOfData”, respectively (38).

## RESULTS AND DISCUSSION

The novelty of the reported protocol lies in the analysis of the 3-D mitochondria-associated signal, in two-photon image stacks that were taken in vivo. Our protocol allows the quantification of endothelial mitochondria-associated signal morphology in the brain microvasculature, the cornerstone of the blood-brain barrier and healthy brain function via the maintenance of a continuous blood flow and nutrient supply (40). Studying the dynamic processes that drive and/or shift the continuous and balanced remodeling of mitochondria and their networks is necessary to understand the connection between morphology/structure/function in health and in different pathologies. Several open-source tools, platforms, and software allow the analysis of mitochondria and mitochondrial networks via quantification of fluorescently labeled organelle networks in cells as well as in 2-D images (41–46). However, these dynamic processes are best observed in their native, physiological environment in real time. Our analysis protocol, coupled with statistical tests, allows the quantification of changes in mitochondria-associated signal morphology recorded in vivo real time. In addition, alterations in the geometric parameters allow us to extrapolate mitochondrial fission and fusion processes or their turnover rate, which can be used to investigate the effects of aging or disease conditions on mitochondrial dynamics. A shift between fission and fusion balance and subsequent change in mitochondrial morphology and/or network as well as the altered bioenergetics have been reported in diabetes (47), obesity (48), embryonic development (49), response to brain injury (50), and in AD (51). In addition, aging-associated changes in the brain vasculature such as increased leakiness, decreased branching, tortuosity, structural remodeling, and decreased barrier function (52, 53) as well as changes in mitochondria (14, 54), and the importance of vascular dysfunction in aging-associated pathologies such as Alzheimer’s disease (55–57) have been well documented. Thus, our analysis protocol in combination with in vitro techniques such as mitochondrial respiration, proteomics, and RNA seq can be used to confirm and correlate in vivo observations with in vitro techniques. Moreover, our analysis protocol combined with targeted vessel imaging allows the analysis of signal geometry in different vessel types or sizes. Although we cannot analyze fine structural changes in two-photon image stacks, the strength of real-time, two-photon imaging can be combined with electron or super resolution microscopy to address mitochondrial membrane structure-related questions or to increase spatial resolution at the submicron scale (51, 58–60).

**Figure 10.**
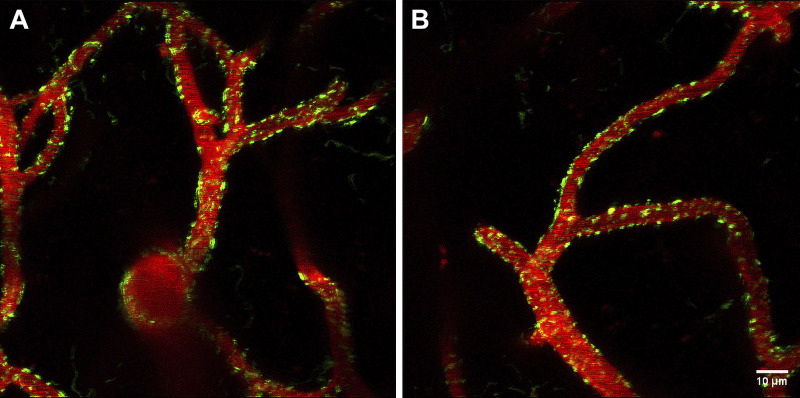
Mitochondria-associated signal morphology. *A* and *B*: representative maximum intensity projected images from a young female mouse indicating heterogeneous mitochondria-associated signal (mito-Dendra2, green). White scale bar denotes 10 μm.

We optimized image processing and thresholding for our images. These parameters may need to be reoptimized upon using our protocol for another cell type or under disease conditions. Some limitations were the use of small animal numbers; although we analyzed many mitochondria within the 3-D stacks, we did not stratify them by vessel type. The data required meticulous interpretation in the context of the experimental setting because the statistical tests detect small changes (*P* < 0.05) that may not be physiologically relevant. Our data showed non-normal distribution when compared with a theoretical Gaussian curve indicated by the histograms (15 images/time points originated from three mice with object numbers *N* = 3,123; *N* = 2,586; *N* = 2,111; *N* = 3,363 for T-0 to T-3, respectively) (Figs. 2 and 3). In addition, data skewness is also demonstrated by the plots of data graphs for M_I analysis method (Figs. 4, 5, 6, 7, and 8); therefore, we provided the median for the different variables. The statistical tests indicate significant differences; however, the effect sizes were small (data not shown) (see appendix, Table A2). In addition, we noted that in most cases significant differences were driven by the T-3 time point. This might be due to the presence of cellular processes or changes that took place by the 12th wk time point making T-3 data different from the other time points. In addition, the presence of a cyclic pattern can be observed in some parameters, which theoretically may reflect hormonal changes in female animals; however, this observation needs to be confirmed in future studies. Because of long reimaging intervals, we were unable to register the images to determine changes in specific object (mitochondrial) morphology because of the dynamic nature of mitochondria; however, future time-lapse imaging using shorter reimaging intervals such as minutes or hours can enable us to determine the fission/fusion turnover rate and morphological changes of specific mitochondria in real time. The visual inspection of representative image stacks of a young female shows heterogeneous morphology (Fig. 10). We speculate that aging will result in a shift in signal morphology and mitochondria will appear more spherical and bulkier in the aged vasculature compared with the young. This expected change is plausible because significantly decreased mitochondrial morphology parameters, including average diameter, average surface area, or the circulatory ratio, have been found in different brain areas of patients with AD when compared with controls (61). In addition, Vue et al. (58, 62) reported aging-associated structure-function changes using high-resolution serial block-face scanning electron microscopy, 3-D reconstruction, and functional assays in cardiac and skeletal muscles. Moreover, Mishra et al. (63) reported an association between mitochondrial morphology visualized by mito-Dendra2 in skeletal muscle fibers and respiratory chain activity. Furthermore, mitochondrial morphology and dynamics assessed via mito-Dendra2 labeling have been investigated in intact arteries and endothelial cells (64), as well as in salivary acinar cells (65).

In conclusion, our analysis protocol, in combination with sensitive statistical tests, is a valuable tool for the assessment of endothelial mitochondria in the brain. We propose that our analysis pipeline will be sensitive and useful in the assessment of changes in mitochondrial, signal-associated morphology during aging or under different pathological conditions. Furthermore, investigating fission-fusion processes in real time will enhance our understanding of chronological events that lead to or drive vascular aging and perhaps dementia.

## DATA AVAILABILITY

The data sets analyzed in the current study are available from the corresponding author on reasonable request.

## GRANTS

This research was supported in part by National Institutes of Health Grants R01AG083567 (to I.R.), 3P30GM145498-02S1 (to I.R.; Principal Investigator, Dr. Jazwinski), R01HL148836 (to D.W.B.), AG075988 (to D.W.B.), and AG063345 (to D.W.B.) and the Louisiana Board of Regents Endowed Chairs for Eminent Scholars program (to D.W.B.).

## DISCLOSURES

No conflicts of interest, financial or otherwise, are declared by the authors.

## AUTHOR CONTRIBUTIONS

C.R.G. and I.R. conceived and designed research; C.R.G., E.N.M., and I.R. analyzed data; C.R.G. and I.R. interpreted results of experiments; C.R.G. and I.R. prepared figures; I.R. drafted manuscript; C.R.G., E.N.M., P.K.C., D.W.B., and I.R. edited and revised manuscript; C.R.G., E.N.M., P.K.C., D.W.B., and I.R. approved final version of manuscript.

## APPENDIX

1) Schematics of data organization methods are shown in Fig. A1.
2) Medians, statistical analyses, and percentiles are shown in Tables A1–A3, respectively.

**Table A1.** Medians for mitochondria-associated signal morphology parameters

		Median			
	Data	T-0	T-1	T-2	T-3
Voxel volume, µm^3^	M_I	0.72	0.72	0.72	0.66
	M_II	0.69	0.66	0.72	0.66
	M_III	0.72	0.66	0.66	0.66
Isosurface volume, µm^3^	M_I	0.47	0.46	0.48	0.39
	M_II	0.47	0.46	0.50	0.41
	M_III	0.50	0.45	0.47	0.39
Surface, µm^2^	M_I	4.17	4.00	4.25	3.58
	M_II	4.20	3.94	4.36	3.61
	M_III	4.20	4.00	4.39	3.61
Length, µm	M_I	2.31	2.24	2.33	2.17
	M_II	2.30	2.24	2.30	2.20
	M_III	2.29	2.23	2.37	2.19
Smallest width of the principal box, µm	M_I	0.82	0.81	0.80	0.82
	M_II	0.81	0.79	0.80	0.81
	M_III	0.80	0.80	0.80	0.84
Largest width of the principal box, µm	M_I	1.22	1.16	1.22	1.12
	M_II	1.23	1.16	1.23	1.11
	M_III	1.21	1.16	1.25	1.13
Corrected lateral width, µm	M_I	0.72	0.72	0.71	0.72
	M_II	0.72	0.72	0.70	0.73
	M_III	0.72	0.72	0.70	0.73
Axial width, µm	M_I	1.10	1.14	1.10	1.15
	M_II	1.10	1.14	1.10	1.14
	M_III	1.10	1.15	1.10	1.14
Roughness sphericity	M_I	0.69	0.72	0.69	0.72
	M_II	0.70	0.71	0.69	0.71
	M_III	0.69	0.72	0.69	0.72
Axial sphericity	M_I	0.51	0.56	0.51	0.60
	M_II	0.53	0.56	0.50	0.59
	M_III	0.53	0.57	0.52	0.58
Axial sphericity of principal box	M_I	0.58	0.61	0.57	0.65
	M_II	0.59	0.60	0.57	0.64
	M_III	0.59	0.62	0.58	0.63
Axial aspect ratio	M_I	2.07	1.82	2.19	1.62
	M_II	2.10	1.83	2.14	1.72
	M_III	2.00	1.73	2.08	1.73
Lateral aspect ratio	M_I	3.25	2.99	3.44	2.76
	M_II	3.15	3.01	3.48	2.85
	M_III	3.21	2.89	3.33	2.86

Medians for mitochondria-associated signal morphology parameters for the consecutive imaging time points (T-0 to T-3) stratified by data organizing methods (M_I to M_III) are shown.

**Table A2.** Statistical analysis and post hoc tests for Method_I by Kruskal–Wallis and MRPP

	*P* Value			*P* Value	
	Kruskal–Wallis	MRPP	Time Points	Kruskal–Wallis DSCF	MRPP Holm–Bonferroni
Voxel volume µm^3^	0.0005	0.0008	T-0 vs. T-1	0.9999	1.0000
			T-0 vs. T-2	0.9991	0.7932
			T-0 vs. T-3	0.0033	0.0032
			T-1 vs. T-2	0.9967	0.7932
			T-1 vs. T-3	0.0081	0.0050
			T-2 vs. T-3	0.0053	0.0316
Isosurface volume, µm^3^	<0.0001	<0.0001	T-0 vs. T-1	0.9997	0.9498
			T-0 vs. T-2	0.9498	0.9498
			T-0 vs. T-3	<0.0001	<0.0001
			T-1 vs. T-2	0.9447	0.9498
			T-1 vs. T-3	<0.0001	<0.0001
			T-2 vs. T-3	<0.0001	<0.0001
Surface, µm^2^	<0.0001	<0.0001	T-0 vs. T-1	0.2227	0.2341
			T-0 vs. T-2	0.7929	0.2685
			T-0 vs. T-3	<0.0001	<0.0001
			T-1 vs. T-2	0.0462	0.1523
			T-1 vs. T-3	<0.0001	<0.0001
			T-2 vs. T-3	<0.0001	<0.0001
Length, µm	<0.0001	<0.0001	T-0 vs. T-1	<0.0001	<0.0001
			T-0 vs. T-2	0.6795	0.1230
			T-0 vs. T-3	<0.0001	<0.0001
			T-1 vs. T-2	<0.0001	<0.0001
			T-1 vs. T-3	<0.0001	<0.0001
			T-2 vs. T-3	<0.0001	<0.0001
Smallest width of principal box, µm	0.0699	0.0008	T-0 vs. T-1	0.8434	0.2642
			T-0 vs. T-2	0.1552	0.0855
			T-0 vs. T-3	0.9637	0.2366
			T-1 vs. T-2	0.6113	0.0471
			T-1 vs. T-3	0.5828	0.2366
			T-2 vs. T-3	0.0609	0.0004
Largest width of principal box, µm	0.1138	0.0007	T-0 vs. T-1	0.7913	0.2376
			T-0 vs. T-2	0.9990	0.5463
			T-0 vs. T-3	0.1642	0.0046
			T-1 vs. T-2	0.7480	0.1012
			T-1 vs. T-3	0.7264	0.5244
			T-2 vs. T-3	0.1722	0.0027
Corrected lateral width, µm	0.0403	0.0062	T-0 vs. T-1	0.9961	0.8029
			T-0 vs. T-2	0.1486	0.0779
			T-0 vs. T-3	0.8728	0.8029
			T-1 vs. T-2	0.2805	0.0165
			T-1 vs. T-3	0.7856	0.8029
			T-2 vs. T-3	0.0212	0.0096
Axial width, µm	<0.0001	<0.0001	T-0 vs. T-1	0.0001	0.0002
			T-0 vs. T-2	0.9996	0.8344
			T-0 vs. T-3	<0.0001	<0.0001
			T-1 vs. T-2	0.0011	0.0006
			T-1 vs. T-3	0.0423	0.0315
			T-2 vs. T-3	<0.0001	<0.0001
Roughness sphericity	<0.0001	<0.0001	T-0 vs. T-1	<0.0001	<0.0001
			T-0 vs. T-2	0.6023	0.1155
			T-0 vs. T-3	<0.0001	<0.0001
			T-1 vs. T-2	<0.0001	<0.0001
			T-1 vs. T-3	0.5227	0.1430
			T-2 vs. T-3	<0.0001	<0.0001
Axial sphericity	<0.0001	<0.0001	T-0 vs. T-1	<0.0001	<0.0001
			T-0 vs. T-2	0.0538	0.0079
			T-0 vs. T-3	<0.0001	<0.0001
			T-1 vs. T-2	<0.0001	<0.0001
			T-1 vs. T-3	<0.0001	<0.0001
			T-2 vs. T-3	<0.0001	<0.0001
Axial sphericity of principal box	<0.0001	<0.0001	T-0 vs. T-1	<0.0001	<0.0001
			T-0 vs. T-2	0.0111	0.0018
			T-0 vs. T-3	<0.0001	<0.0001
			T-1 vs. T-2	<0.0001	<0.0001
			T-1 vs. T-3	<0.0001	<0.0001
			T-2 vs. T-3	<0.0001	<0.0001
Axial aspect ratio	<0.0001	<0.0001	T-0 vs. T-1	<0.0001	<0.0001
			T-0 vs. T-2	0.3525	0.1571
			T-0 vs. T-3	<0.0001	<0.0001
			T-1 vs. T-2	<0.0001	<0.0001
			T-1 vs. T-3	<0.0001	<0.0001
			T-2 vs. T-3	<0.0001	<0.0001
Lateral aspect ratio	<0.0001	<0.0001	T-0 vs. T-1	<0.0001	<0.0001
			T-0 vs. T-2	0.0052	0.0017
			T-0 vs. T-3	<0.0001	<0.0001
			T-1 vs. T-2	<0.0001	<0.0001
			T-1 vs. T-3	<0.0001	<0.0001
			T-2 vs. T-3	<0.0001	<0.0001

DSCF, Dwass–Steel–Critchlow–Fligner post hoc pairwise comparisons; MRPP, multiresponse permutation procedure; T-0 to T-3, 4 consecutive imaging time points.

**Table A3.** Percentiles for Method_I data stratified by consecutive imaging time points

		Percentiles			
	Time Points	10	25	75	90
Voxel volume, µm^3^	T-0	0.36	0.48	1.21	2.11
	T-1	0.36	0.48	1.27	2.11
	T-2	0.36	0.48	1.21	1.99
	T-3	0.36	0.42	1.15	1.99
Isosurface volume, µm^3^	T-0	0.16	0.26	0.97	1.96
	T-1	0.15	0.25	1.00	1.84
	T-2	0.17	0.26	0.97	1.76
	T-3	0.13	0.22	0.82	1.60
Surface, µm^2^	T-0	1.98	2.67	7.13	11.59
	T-1	1.84	2.58	7.06	10.91
	T-2	2.02	2.81	7.20	11.28
	T-3	1.66	2.31	6.10	10.18
Length, µm	T-0	1.08	1.85	3.22	4.18
	T-1	1.06	1.47	3.14	4.07
	T-2	1.10	2.04	3.24	4.13
	T-3	1.04	1.31	2.92	3.58
Smallest width of principal box, µm	T-0	0.57	0.67	1.01	1.30
	T-1	0.55	0.65	1.00	1.33
	T-2	0.57	0.66	1.00	1.26
	T-3	0.56	0.66	1.00	1.36
Largest width of principal box, µm	T-0	0.93	1.00	1.66	2.15
	T-1	0.97	1.00	1.68	2.20
	T-2	0.92	1.00	1.65	2.09
	T-3	0.99	1.00	1.66	2.15
Corrected lateral width, µm	T-0	0.50	0.56	0.91	1.20
	T-1	0.49	0.55	0.93	1.23
	T-2	0.50	0.55	0.89	1.14
	T-3	0.50	0.56	0.93	1.21
Axial width, µm	T-0	0.66	0.85	1.34	1.83
	T-1	0.68	0.90	1.39	1.92
	T-2	0.65	0.84	1.36	1.81
	T-3	0.74	0.93	1.41	1.95
Roughness sphericity	T-0	0.56	0.62	0.78	0.86
	T-1	0.58	0.64	0.81	0.89
	T-2	0.56	0.62	0.77	0.85
	T-3	0.57	0.64	0.81	0.89
Axial sphericity	T-0	0.37	0.43	0.66	0.81
	T-1	0.38	0.45	0.71	0.82
	T-2	0.36	0.42	0.64	0.79
	T-3	0.42	0.49	0.75	0.84
Axial sphericity of principal box	T-0	0.43	0.50	0.70	0.82
	T-1	0.44	0.52	0.73	0.82
	T-2	0.42	0.49	0.68	0.80
	T-3	0.48	0.55	0.77	0.86
Axial aspect ratio	T-0	1.10	1.40	3.14	4.17
	T-1	1.06	1.29	2.73	3.81
	T-2	1.11	1.48	3.13	4.16
	T-3	1.00	1.20	2.38	3.30
Lateral aspect ratio	T-0	1.75	2.30	4.23	5.40
	T-1	1.61	2.12	4.05	5.25
	T-2	1.89	2.44	4.32	5.48
	T-3	1.51	1.99	3.73	4.54

T-0 to T-3, four consecutive imaging time points.
